# Significant intraocular pressure associated with open-angle glaucoma: Korea National Health and Nutrition Examination Survey 2010-2011

**DOI:** 10.1371/journal.pone.0235701

**Published:** 2020-07-16

**Authors:** Ko Eun Kim, Joon Mo Kim, Jungmin Lee, Mi-Yeon Lee, Ki Ho Park

**Affiliations:** 1 Department of Ophthalmology, Nowon Eulji Medical Center, Eulji University, Seoul, Republic of Korea; 2 Department of Ophthalmology, Kangbuk Samsung Hospital, Sungkyunkwan University School of Medicine, Seoul, Republic of Korea; 3 Division of Biostatistics, Department of R&D Management, Kangbuk Samsung Hospital, Sungkyunkwan University School of Medicine, Seoul, Republic of Korea; 4 Department of Ophthalmology, Seoul National University Hospital, Seoul National University College of Medicine, Seoul, Republic of Korea; Bascom Palmer Eye Institute, UNITED STATES

## Abstract

**Objectives:**

To investigate significant intraocular pressure (IOP) levels associated with the risk of open-angle glaucoma (OAG) in the treatment-naïve Korean population.

**Methods:**

Participants ≥20 years of age in Korea National Health and Nutrition Examination Survey 2010–2011 were divided into two groups, those with higher and lower IOP values, compared with the reference IOP value. We compared the risk of OAG in each group using regression analyses. The IOP value that yielded the highest statistical significance was determined as an IOP significantly associated with the OAG risk.

**Results:**

A total of 7,650 participants (7,292 control, 358 OAG) were included. The mean IOP was significantly higher in OAG group (14.4 ± 2.9 mmHg), compared to control group (13.9 ± 2.7 mmHg, *P* = 0.022). In association with an increased risk of OAG, the significant IOP value was 18 mmHg (Odds ratio [OR] = 1.79, 95% confidence interval [CI] 1.14–2.80, *P* = 0.011). Additionally, sex-difference was identified and they were 19 mmHg (OR = 2.79, 95% CI 1.27–6.16, *P* = 0.011) in men and 18 mmHg (OR = 2.65, 95% CI 1.32–5.33, *P* = 0.006) in women. The IOP values associated with significantly decreased risk of glaucoma were determined to be 14 mmHg in men (OR = 0.68, 95% CI 0.47–0.99, *P* = 0.042) and 16 mmHg in women (OR = 0.47, 95% CI 0.27–0.81, *P* = 0.007).

**Conclusions:**

In consideration of the risk to benefit ratio, the reference IOP level for screening or setting the target IOP for treatment could be considered different from traditional 21 mmHg in Korean population.

## Introduction

Glaucoma is a chronic, progressive optic neuropathy characterized by change in the optic nerve head and corresponding visual field loss [1]. High intraocular pressure (IOP) has been considered to be one of the most important risk factors for developing glaucoma [1–3]. The normal IOP range, defined as the mean IOP within 2 standard deviations (SDs) has been considered to be 10–21 mmHg [3]. Thus, traditional criterion for an “abnormal” or “high” IOP has been regarded as an IOP greater than 21 mmHg, an IOP level that exceeds the 97.5^th^ percentile value. However, there are limitations to this criterion for assessing the risk of glaucoma in the real world.

A number of previous studies reported that the IOP level in the general population does not represent a Gaussian distribution [3]. In addition, the IOP distribution curves in glaucomatous and control eyes overlap to a great extent, and thus, they cannot be simply divided by one definite IOP level. Moreover, in Asian countries, OAG patients with a baseline IOP of ≤21 mmHg are more prevalent than those with a baseline IOP of >21 mmHg [4,5], which suggests different population groups may require different IOP criteria. In this regard, an abnormal IOP value of >21 mmHg, may have limited clinical relevance for a generalized application of screening eyes at risk of glaucoma or ocular hypertension. These differences suggest that the normal IOP range, as well as the significant IOP value associated with the risk of glaucoma should be applied distinctively, with consideration of baseline IOP characteristic values in various populations. This is also important in the perspective of establishing a target IOP and evaluating the efficacy of glaucoma treatment.

In light of these, the purpose of this study was to investigate the IOP level that is significantly associated with the risk of glaucoma in treatment-naïve population based on the Korea National Health and Nutrition Examination Survey (KNHANES) 2010–2011 data. Moreover, we examined the range and distribution of IOP in healthy and OAG groups.

## Materials and methods

The KNHANES is a nationwide population-based cross-sectional survey of the South Korean population that is conducted by the Korea Centers for Disease Control and Prevention and the Korean Ministry of Health and Welfare [5–7]. Using a multistage, stratified, probability-clustered sampling method and weighting scheme, the KNHANES provides estimated health statistics that are representative of the civilian, non-institutionalized South Korean population. This survey adhered to the tenets of the Declaration of Helsinki for human research, and all participants provided written informed consent. The survey protocol was approved by the Institutional Review Board of the Korea Center for Disease Control and Prevention. Since all of the KNHANES data are anonymized, the Institutional Review Board of Kangbuk Samsung Hospital agreed that this study was exempt from requiring subject approval.

### Study design and examinations

All subjects had a health interview survey that included standardized questionnaires on demographic variables, as well as current and past medical history, health-influencing behaviors, and socioeconomic status. They also had a health examination survey that included physical and ophthalmologic examinations.

The comprehensive ophthalmologic examinations were performed by ophthalmologists trained by the Korean Ophthalmology Society National Epidemiologic Survey Committee. After a health interview that included previous ophthalmic disease-related history, a visual acuity by Snellen chart, the IOP by Goldmann applanation tonometry (GAT), and spherical equivalent (SE), using an automatic refractometer (KR-8800; Topcon, Tokyo, Japan), were measured. A slit-lamp examination (Haag-Streit model BQ-900; Haag-Streit AG, Koeniz, Switzerland) was performed to evaluate the anterior segment and peripheral anterior chamber depth. A peripheral anterior chamber depth of >1/4 peripheral corneal thickness by the Van Herick method was defined as an open angle. Retinal examinations were performed by obtaining a nonmydriatic digital fundus photograph (TRCNW6S; Topcon) of each eye from all of the subjects in a dark room. Visual field testing was performed using the frequency doubling technology (FDT; Humphrey Matrix FDT perimetry; Carl Zeiss Meditec, Inc., Dublin, CA, USA) with the N30-1 screening program on subjects who showed elevated IOP (≥22 mm Hg) or glaucomatous optic discs.

### Glaucoma diagnosis

A glaucoma diagnosis was made based on the fundus photography and FDT perimetry findings, according to the International Society of Geographical and Epidemiological Ophthalmology criteria [8] and the findings from previous studies [5,7]. After the preliminary grading based on the glaucoma reading by a committee comprised of glaucoma specialists, the detailed grading was independently performed by another group of glaucoma specialists who were blind to the participants’ other information. Any discrepancies between the preliminary and detailed grading were adjudicated by a third group of glaucoma specialists. The glaucoma group was defined based on the ISGEO criteria category I or II [8]. Category I requires a visual field defect consistent with glaucoma and either a vertical cup-to-disc ratio (VCDR) of ≥0.7 (97.5th percentile) or VCDR asymmetry of ≥0.2 between the right and left eyes (97.5th percentile). Category II indicates that the visual field results are not definitive, requiring a VCDR of ≥0.9 (99.5th percentile) or VCDR asymmetry of ≥0.3 (99.5th percentile).

### Systemic variable definition

Physical measurements included height, weight, systolic and diastolic blood pressures, waist circumference, and body mass index (BMI, the ratio of weight divided by height squared). A morning blood sample was collected after at least 12 hours of fasting. Impaired fasting glucose was defined as fasting blood glucose between 100 mg/dl and 126 mg/dl. Diabetes mellitus (DM) was defined as a fasting glucose value of ≥126 mg/dl, use of oral hypoglycemic agents or insulin, or a history of DM. Prehypertension was defined as systolic blood pressure between 120 mmHg and 140 mmHg or diastolic blood pressure between 80 mmHg and 90 mmHg. Hypertension was defined as systolic blood pressure greater than 140 mmHg, diastolic blood pressure greater than 90 mmHg, or use of antihypertensive medication.

### Statistical analysis

All data were analyzed using IBM SPSS Statistics for Windows, version 24.0 (IBM Corp., Armonk, NY) to account for the complex sampling design. Strata, sampling units, and sampling weights were used to obtain point estimates and standard errors (SEs) of the mean. All data were analyzed with weighted data, and the SEs of mean population estimates were calculated by Taylor linearization methods. Participant characteristics were summarized as means and SEs for continuous variables and as frequencies and percentages for categorical variables. Demographic information and clinical parameters were compared between groups using the Pearson chi-square test for categorical variables and the general linear model for continuous variables. The right eye was used for controls and bilateral glaucoma patients, and the affected eye for monocular glaucoma patients.

Participants were divided into two subgroups, those with higher versus lower IOP values compared with each reference IOP value. We analyzed the risk of OAG (presented as an odds ratio [OR] with 95% confidence interval [CI]) for each group using univariate and multivariate regression analyses adjusted for age, sex, DM, systemic hypertension, BMI, serum cholesterol. The optimal reference IOP value that yielded the highest statistical significance was then determined as IOP level that was significant for the increased or decreased risk of OAG.

## Results

During 2010–2011, a total of 12,356 non-institutionalized South Koreans ≥20 years of age participated in the KNHANES. Exclusion of 3,147 subjects who did not undergo ophthalmic examinations left 9,209 eligible subjects. Participants were excluded from the study if they had any history of cataracts (n = 93), retinal (n = 48) or refractive surgeries (n = 376), showed evidence of retinal detachment or age-related macular degeneration (n = 22), or had any missing data (n = 927). Participants who were diagnosed and treated for glaucoma were also excluded (n = 93). Finally, a total of 7,650 participants (7,292 controls and 358 OAG patients) were included in the analysis.

**Fig 1:**
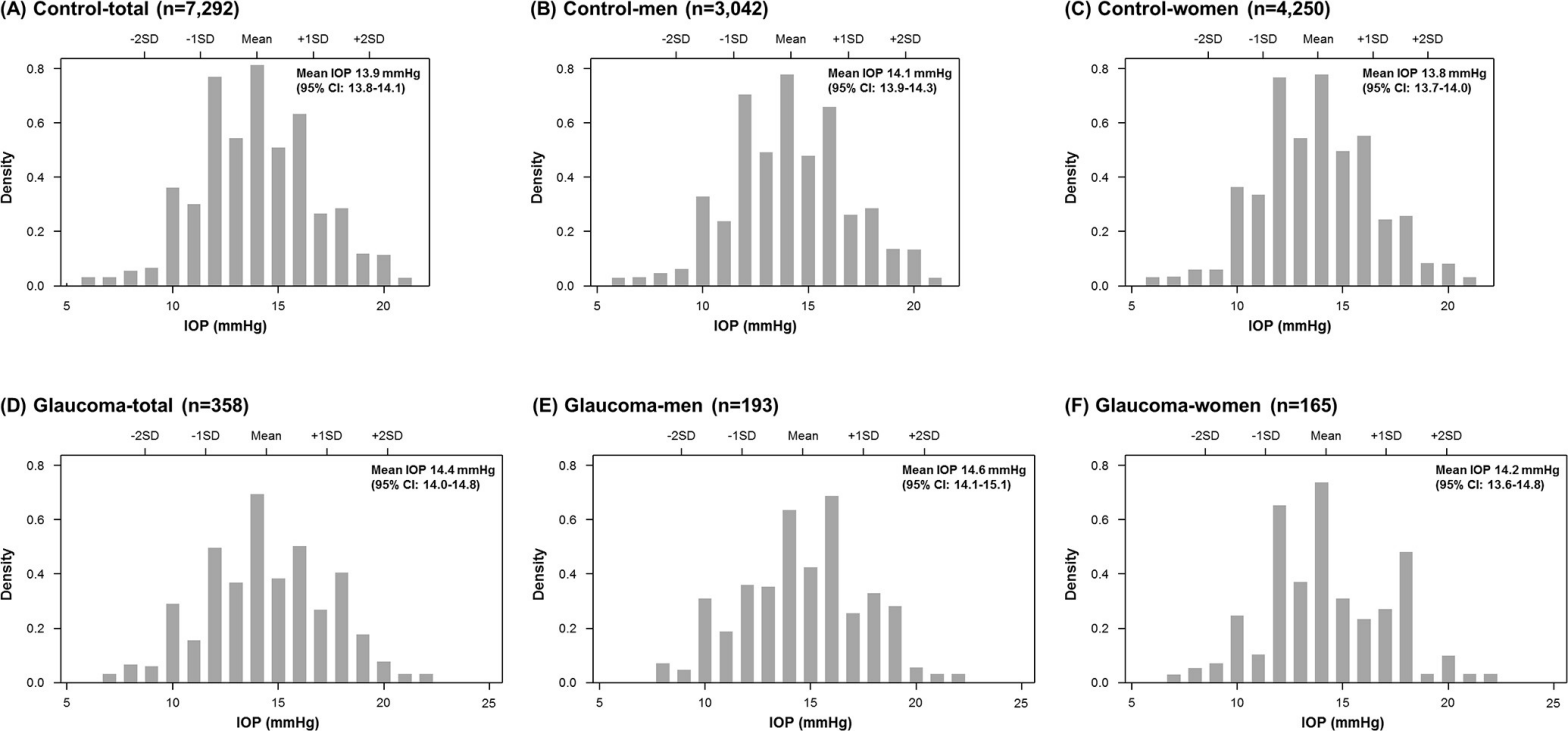
Density histograms showing intraocular pressure (IOP) distributions in (A, B, C) control and (D, E, F) glaucoma groups. The mean IOP was significantly higher in the OAG group (14.4 ± 2.9 mmHg, range 7–22 mmHg) compared to the control group (13.9 ± 2.7 mmHg, range 6–21 mmHg, *P* = 0.022). However, no significant difference was found in separate analyses of men and women. SD = standard deviation; CI = confidence interval.

**Fig 2:**
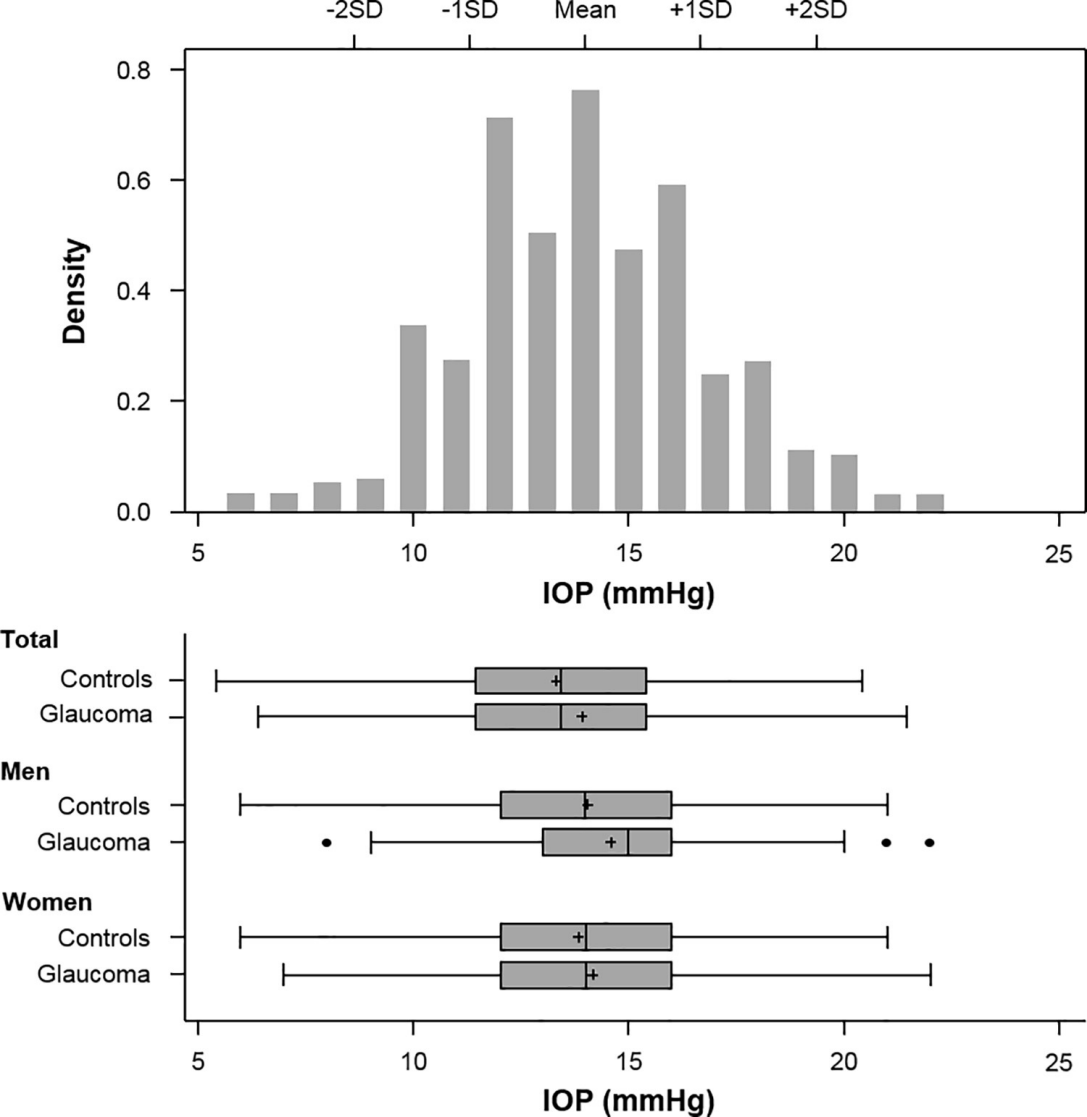
The distribution of intraocular pressure (IOP) in control and open-angle glaucoma (OAG) groups. SD = standard deviation.

The OAG group had significantly older mean age values and higher rates of systemic hypertension and DM compared to the control group. A separate analysis in women revealed that the OAG group had significantly larger waist circumferences and BMI values, higher total cholesterol and triglycerides, and diastolic blood pressure than the control group (Table 1). Older age (*P* <0.001), male (*P* = 0.012), and higher IOP (*P* = 0.021) were significantly associated with OAG, but hypertension (*P* = 0.278) and diabetes mellitus (*P* = 0.343) were not after univariate and multivariate logistic regression analyses. The mean IOP was significantly higher in the OAG group (14.4 ± 2.9 mmHg, range 7–22 mmHg) compared to the control group (13.9 ± 2.7 mmHg, range 6–21 mmHg, *P* = 0.022, Fig 1). The IOP measurement distribution showed a right-sided skew with a skewness of 0.16 (SE 0.03) and -0.02 (SE 0.13) and kurtosis of 2.68 (SE 0.03) and 2.54 (SE 0.11) in the control and OAG groups, respectively. The IOP ranges within the mean ± 2SD were 8.7–19.3 mmHg in the control group and 8.7–20.2 mmHg in the glaucoma group (Fig 2).

**Table 1:** Baseline characteristics of participants included in the Korean National Health and Nutrition Examination Survey 2010–2011.

Variables	Male (n = 3,235)			Female (n = 4,415)		
	Controls (n = 3,042)	Glaucoma (n = 193)	*P*	Controls (n = 4,250)	Glaucoma (n = 165)	*P*
Age (yrs)	41.4 (0.4)	49.5 (1.3)	**<0.001**	43.1 (0.3)	52.2 (1.8)	**<0.001**
Intraocular pressure (mmHg)	14.1 (0.1)	14.6 (0.2)	0.059	13.8 (0.1)	14.20 (0.3)	0.216
Smoker (%)	49.4 (1.2)	45.4 (4.3)	0.376	6.62 (0.51)	7.02 (2.35)	0.857
Drinker (%)	79.9 (1.0)	76.0 (4.0)	0.314	44.3 (1.0)	39.4 (5.0)	0.341
Hypertension (%)			**0.023**			**<0.001**
Pre-hypertension	30.4 (1.1)	28.3 (3.9)		17.1 (0.7)	31.5 (4.5)	
Hypertension	22.8 (1.0)	33.4 (4.2)		16.7 (0.7)	26.1 (9.9)	
Diabetes mellitus (DM, %)			**0.007**			**0.044**
DM	7.7 (0.6)	16.0 (3.4)		5.5 (0.4)	10.6 (2.9)	
Pre-DM	19.5 (1.0)	19.4 (3.8)		12.2 (0.6)	14.5 (3.2)	
Body height (cm)	171.5 (0.2)	169.6 (0.7)	**0.005**	158.0 (0.1)	155.0 (0.6)	**<0.001**
Waist circumference (cm)	84.5 (0.2)	83.4 (0.7)	0.137	77.6 (0.2)	80.3 (1.1)	**0.013**
Body mass index (kg/m^2^)	24.2 (0.1)	23.6 (0.2)	**0.007**	23.2 (0.1)	24.1 (0.4)	**0.021**
Glucose (mg/dL)	97.4 (0.5)	101.7 (2.7)	0.124	93.0 (0.3)	96.7 (1.5)	**0.016**
HDL (mg/dL)	49.8 (0.3)	48.8 (1.3)	0.398	56.4 (0.3)	54.3 (1.1)	0.071
LDL (mg/dL)	114.2 (1.1)	109.3 (5.5)	0.390	110.4 (1.1)	115.2 (7.1)	0.495
Total cholesterol (mg/dL)	189.0 (0.9)	186.7 (4.6)	0.635	186.5 (0.8)	194.8 (3.5)	**0.020**
Triglycerides (mg/dL)	158.1 (3.1)	170.5 (21.1)	0.557	105.8 (1.4)	123.8 (6.1)	**0.004**

Data are weighted means with the standard error in parentheses for continuous variable and frequencies and percentages in parentheses for categorical variables.

*P* values resulted from the general linear model for continuous variables.

The risk of glaucoma significantly increased as the reference IOP level was set at 18 mmHg (OR = 1.79, 95% CI 1.14–2.80, *P* = 0.011, Table 2). The IOP value that was significant for an increased risk of glaucoma was calculated as 19 mmHg in men (OR = 2.79, 95% CI 1.27–6.16, *P* = 0.011) and 18 mmHg in women (OR = 2.65, 95% CI 1.32–5.33, *P* = 0.006). In comparison, the IOP values associated with a significantly decreased (protective) risk of glaucoma were determined to be 14 mmHg in men (OR = 0.68, 95% CI 0.47–0.99, *P* = 0.042) and 16 mmHg in women (OR = 0.47, 95% CI 0.27–0.81, *P* = 0.007).

**Table 2:** The risk of open-angle glaucoma based on each reference intraocular pressure (IOP) level.

Reference IOP level (mmHg)	Total (n = 7,650)			Men (n = 3,235)			Women (n = 4,415)		
	Frequency (n, %)	OR (95% CI)	*P*	Frequency (n, %)	OR (95% CI)	*P*	Frequency (n, %)	OR (95% CI)	*P*
≤ 14	4,635 (59.9)	0.78 (0.58–1.04)	0.092	1,880 (57.1)	0.68 (0.47–0.99)	**0.042**	2,755 (62.6)	0.95 (0.63–1.43)	0.810
15	818 (10.4)	0.85 (0.55–1.32)	0.472	356 (10.3)	0.95 (0.55–1.65)	0.856	462 (10.5)	0.72 (0.39–1.34)	0.300
16	936 (13.0)	0.98 (0.65–1.50)	0.938	408 (14.3)	1.37 (0.83–2.26)	0.222	528 (11.7)	0.47 (0.27–0.81)	**0.007**
17	421 (5.5)	1.25 (0.71–2.20)	0.437	186 (5.7)	1.11 (0.50–2.46)	0.802	235 (5.3)	1.49 (0.68–3.26)	0.323
18	465 (6.0)	1.79 (1.14–2.80)	**0.011**	215 (6.2)	1.30 (0.72–2.33)	0.378	250 (5.8)	2.65 (1.32–5.33)	**0.006**
19	173 (2.5)	1.88 (0.92–3.84)	0.083	83 (3.2)	2.79 (1.27–6.16)	**0.011**	90 (1.8)	0.35 (0.05–2.63)	0.308
20	163 (2.3)	0.83 (0.30–2.30)	0.723	88 (2.8)	0.51 (0.10–2.67)	0.424	75 (1.8)	1.60 (0.47–5.44)	0.450
≥ 21	39 (0.4)	2.64 (0.88–7.91)	0.083	19 (0.5)	2.55 (0.65–10.02)	0.179	20 (0.4)	2.86 (0.49–16.63)	0.241

OR = odds ratio; CI = confidence interval.

Boldface values are significant at *P* < 0.05.

## Discussion

The main pathophysiology of glaucoma has long been attributed to a high IOP of more than 21 mmHg, which represents an IOP greater than the 97.5^th^ percentile value in the general population. However, the IOP value reflecting the risk of OAG has not been sufficiently investigated with evidence-based research. Moreover, a clear basis for the upper pressure threshold for glaucomatous damage has not yet been defined for different ethnicities. In this regard, based on our population-based survey, we investigated the clinically meaningful IOP values associated with the risk of glaucoma, independent of age, sex, and systemic variables including DM, systemic hypertension, BMI, and serum cholesterol. The significant IOP level that indicated a higher risk of glaucoma was 18 mmHg in the treatment-naïve Korean population based on the KNHANES 2010–2011. Moreover, a sex-difference was identified, indicating risk values of 19 mmHg in men and 18 mmHg in women. Therefore, we concluded that at least in the Korean population, the reference IOP level for screening or setting the target IOP for treatment cannot always be set as 21 mmHg. These further indicate that in populations with higher proportion of patients with lower untreated IOP, different IOP criteria can be considered and evaluating the risk of glaucoma cannot be solely dependent on the IOP itself.

A number of previous population-based studies have reported IOP measurements that were within a mean ± SD and corresponding ranges for a healthy population. For example, the values were 15.4 ± 3.3 in the Beaver-Dam study [9], 13.6 ± 3.4 mmHg in studies in Central India, 14.3 ± 3.3 mmHg in South India, and 13.6 ± 3.8 mmHg in the Ural Eye and Medical Study (Russian population; [10]. Similarly, the mean IOP was 13.9 ± 2.7 mmHg and the range within the 97.5^th^ percentile was 8.0–20.0 mmHg in the healthy population of the KNHANES 2010–2011. Then, the upper threshold of abnormal IOP level in this study would be 20 mmHg, when applying the traditional concept. However, we speculated that it would be more reasonable to set the contemporary definition of abnormal IOP as a “clinically meaningful IOP”, which would be significantly associated with an increased risk of glaucoma. As a result, the values in this study were 19 mmHg in men and 18 mmHg in women in the Korean population, after adjusting for important systemic variables.

A higher IOP has been associated with a higher likelihood of developing glaucoma. However, in the present study, the risk of glaucoma did not continuously increase with corresponding increases in the IOP elevation. This is partially consistent with the results from the Namil study, which was another epidemiological study conducted in South Korea [11]. Although the Namil study presented a general trend of increasing POAG prevalence in subjects with high IOP, the prevalence did not reach the highest point in subjects with the highest IOP. These results are also in agreement with Tajimi study [12] from Japan and Handan study [13] from China, where up to 90% of OAG patients had an IOP ≤21 mmHg. Thus, we speculate that these results are attributable to a large proportion of subjects with an IOP of ≤21 mmHg in Korea [5], despite the possibility of insufficient statistical power due to the low frequency of patients with an IOP >21 mmHg identified in the KNHANES.

The main purpose of our study was to investigate clinically meaningful IOP values that could suggest the risk of glaucoma, based on our population-based survey. Although our results may have limitations in representing the whole population, these are important for the following reasons. First, the clinically meaningful IOP can be important for establishing the appropriate IOP level for screening glaucoma or ocular hypertension. Although the IOP cannot be a standalone screening tool for glaucoma [14], a rationale is needed to identify IOP measurements that indicate potential glaucoma development. Currently, the upper limit of the normal IOP level worldwide has been set as 21 mmHg, but this criterion may require population-specific revisions, especially for those with a large proportion of glaucoma patients that have lower pre-treatment IOP values. Second, our results can provide guidance for determining the appropriate amount of treatment to reduce the IOP as well as insight into optimal levels that will not increase the likelihood of glaucoma development. In an advanced glaucoma intervention study [15], patients were classified into 3 groups according to IOP levels of 14, 14.5, and 17.5 mmHg and the conclusion was that not only lowering IOP, but maintaining an IOP less than 17.5 mmHg could effectively lower the probability of glaucoma progression. Although the present study was a cross-sectional study, we believe that our data can provide additional insight into the target IOP to be considered as below 18 mmHg and furthermore, less than 14 mmHg for the significantly beneficial effects. This information can also be considered when evaluating the effectiveness of the glaucoma treatment. Third, the primary challenge for initiating glaucomatous damage, especially for those with lower baseline IOP, has been associated with a low threshold for stress tolerance at a certain pressure level rather than the absolute IOP level [16,17]. In addition, the threshold for stress tolerance can differ, depending on various factors including age, sex, and ethnicity. Since the majority of OAG patients in Korea have lower baseline IOP values, we speculated that different pressure criteria would potentially provide new insights for clinicians to better understand such thresholds in Koreans.

Studies have reported different results on the association between IOP and sex: some studies have reported a higher IOP in women than in men [9,10,18,19], and others have reported opposite results [5,20–23]. Based on the KNHANES from 2009–2010, the mean IOP was significantly higher in men than in women and the higher IOP was also significantly correlated with male gender after multivariate analysis [21]. Another study that used a large-scale database of Korean subjects (n = 155,198) also reported the same trend [20]. These studies may account for the higher IOP level in men (19 mmHg) compared with the level for women (18 mmHg) in the present study. Sex-hormone related factors such as the IOP-lowering effect of estrogen, and the IOP increase associated with a relative increase of testosterone levels, in addition to genetic factors, have been suggested as possible mechanisms for the sex-associated IOP differences [24–26]. However, these results remain controversial as conflicting results have been reported in different studies, depending on the covariate adjustment. Therefore, further investigations are needed to elucidate the mechanisms for sex-associated IOP differences.

Several limitations should be considered when interpreting our study. First, the KNHANES had a single IOP measurement, which limits our ability to explore the association between the peak or fluctuating IOP and the risk of glaucoma. Second, the present study was based on treatment-naïve patients. This may have resulted the low frequency of patients with an IOP >21 mmHg. However, the information on the baseline IOP was unavailable from treated glaucoma patients (n = 93), and thus, we speculated that they should not be included in the present study. Third, the FDT was used for the functional examination, which does not meet the standard criteria for a glaucoma diagnosis. Nevertheless, the FDT is a fast, reliable, large-scale screening method frequently used in population-based studies. Moreover, since it can detect glaucomatous visual field defects earlier than the standard automated perimetry, it was optimal for our study to ensure that patients at risk of glaucoma were included [27]. Lastly, the angle was assessed using the Van Herick methods and not a gonioscopic examination, thus subjects with angle closure may have been included in our OAG population. Despite these limitations, our study population had a relatively large sample size and a high participation rate, which was representative of the whole population in South Korea.

In conclusion, the IOP value associated with a significantly increased risk of OAG was 18 mmHg; the value was 19 mmHg in men and 18 mmHg in women. Therefore, in consideration of the risk to benefit ratio, the reference IOP level for screening or setting the target IOP for treatment could be considered different from traditional 21 mmHg in Korean population. Additional clinical studies are needed to further elucidate applications of our results in Koreans.
